# *APOE* genotype, hypertension severity and outcomes after intracerebral haemorrhage

**DOI:** 10.1093/braincomms/fcz018

**Published:** 2019-09-14

**Authors:** Alessandro Biffi, Meredith P Murphy, Patryk Kubiszewski, Christina Kourkoulis, Kristin Schwab, Mahmut Edip Gurol, Steven M Greenberg, Anand Viswanathan, Christopher D Anderson, Jonathan Rosand

**Affiliations:** 1 Henry and Allison McCance Center for Brain Health, MGH, Boston, MA, USA; 2 Division of Behavioral Neurology, Department of Neurology, MGH, Boston, MA, USA; 3 Division of Neuropsychiatry, Department of Psychiatry, MGH, Boston, MA, USA; 4 Hemorrhagic Stroke Research Program, J. Philip Kistler Stroke Research Center, MGH, Boston, MA, USA; 5 Center for Genomic Medicine, Massachusetts General Hospital (MGH), Boston, MA, USA; 6 Division of Neurocritical Care and Emergency Neurology, Department of Neurology, MGH, Boston, MA, USA; 7 Program in Medical and Population Genetics, Broad Institute of Harvard and MIT, Cambridge, MA, USA

**Keywords:** intracerebral haemorrhage, hypertension, APOE

## Abstract

Intracerebral haemorrhage in the elderly is a severe manifestation of common forms of cerebral small vessel disease. Nearly 60% of intracerebral haemorrhage survivors will develop clinical manifestations of small vessel disease progression including recurrent haemorrhage, ischaemic stroke, dementia, late-life depression and gait impairment within 5 years. Blood pressure measurements following intracerebral haemorrhage are strongly associated with this risk. However, aggressive blood pressure lowering in the elderly carries substantial risks. In order to determine whether there might be an opportunity to select individuals at the highest risk for small vessel disease progression for aggressive blood pressure reduction, we investigated whether *APOE* gene variants ɛ2/ɛ4 modify the association between blood pressure and small vessel disease clinical progression after intracerebral haemorrhage. We conducted a single-centre longitudinal study at a tertiary care referral centre (Massachusetts General Hospital in Boston, MA, USA), analysing 716 consecutive survivors of acute intracerebral haemorrhage, enrolled from January 2006 to December 2016. We conducted research interviews at the time of enrolment and obtained *APOE* genotypes from peripheral venous blood samples. We followed patients longitudinally by means of validated phone-based research encounters, aimed at gathering measurements of systolic and diastolic blood pressure, as well as information on small vessel disease clinical outcomes (including recurrent haemorrhage, incident ischaemic stroke, incident dementia, incident depression and incident gait impairment). *APOE* ε4 and systolic blood pressure were associated with the risk of recurrent haemorrhage, ischaemic stroke and post-haemorrhage dementia, depression and gait impairment (all *P* < 0.05). *APOE ε4* and systolic blood pressure interacted to increase the risk of recurrent haemorrhage, ischaemic stroke, dementia and gait impairment (all interaction *P* < 0.05). Among patients with elevated blood pressure following intracerebral haemorrhage (average systolic blood pressure 120–129 mmHg and diastolic blood pressure <80 mmHg) only those with one or more *APOE* ε4 copies were at increased risk for one or more small vessel disease outcomes (hazard ratio = 1.97, 95% confidence interval 1.17–3.31). Among haemorrhage survivors with hypertension (stage 1 and beyond) *APOE* genotype also stratified risk for all small vessel disease outcomes. In conclusion, *APOE* genotype modifies the already strong association of hypertension with multiple small vessel disease clinical outcomes among intracerebral haemorrhage survivors. These data raise the possibility that genetic screening could inform blood pressure treatment goals in this patient population.

## Teaser

Intracerebral haemorrhage is a severe manifestation of cerebral small vessel disease, alongside ischaemic stroke, dementia, depression and gait impairment. We found that systolic blood pressure and *APOE* ε4 interact to increase risk of small vessel disease disorders. Genetic screening may, therefore, inform hypertension treatment goals for intracerebral haemorrhage survivors.

## Graphical Abstract

**Graphical Abstract:**
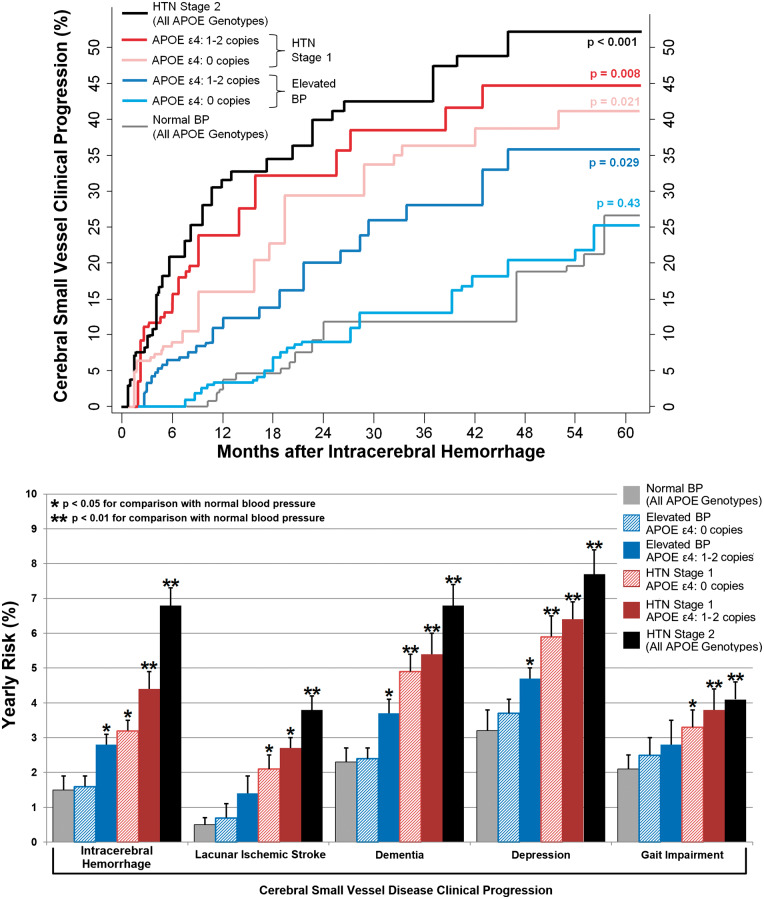

## Introduction

Intracerebral haemorrhage (ICH) is the most severe form of stroke, accounting for 10–15% of all acute cerebrovascular events, and for ∼50% of stroke-related mortality and disability (Qureshi *et al.*, 2001; Poon *et al.*, 2014). Most spontaneous ICH cases are the acute manifestation of age-related cerebral small vessel disease (SVD; Pantoni, 2010; Biffi and Greenberg, 2011). ICH survivors are, therefore, at high risk for all manifestations of progressive SVD: recurrent ICH, ischaemic stroke (especially small vessel infarcts), cognitive impairment, late-life depression and gait impairment (Pantoni, 2010; Benedictus *et al.*, 2015; Biffi *et al.*, 2016; Moulin *et al.*, 2016). The *APOE* gene has been robustly associated with SVD and with ICH risk; indeed, *APOE* variants ɛ2 and ɛ4 represent by far the most potent genetic risk factors for ICH (Greenberg *et al.*, 1995; Tzourio *et al.*, 2008; Biffi *et al.*, 2010*b*).

While blood pressure (BP) control is widely advocated as effective for reducing ICH risk, the optimal degree of BP reduction remains controversial. Published ICH management guidelines recommend achieving goals of Systolic BP (SBP) <130 mmHg and Diastolic BP (DBP) <80 mmHg for secondary prevention (Hemphill *et al.*, 2015). However, findings from randomized trials and a large meta-analysis suggest that individuals at high risk for cardiovascular diseases, as ICH survivors often are, benefit from achieving normal BP (i.e. SBP < 120 and DBP < 80 mmHg; Ettehad *et al.*, 2016; Group *et al.*, 2015). Indeed, the revised ACC/AHA guidelines recently proposed more stringent BP control goals for the general population (Whelton *et al.*, 2018). We ourselves reported increased ICH recurrence risk among individuals with average SBP 120–129 mmHg (Biffi *et al.*, 2015). On the other hand, pharmacological BP reduction in the elderly (the population most at risk for ICH) has been associated with increased risk of ischaemic stroke, cognitive impairment and gait impairment/falls (Eigenbrodt *et al.*, 2000; Hyman and Taffet, 2009; Rose *et al.*, 2010; Butt and Harvey, 2015).

Because of substantial variation in ICH risk based on *APOE* genotype, this genetic information may be of assistance in guiding BP management among ICH survivors. We, therefore, sought to test whether *APOE* genetic variation influences the association between BP and ICH recurrence risk, as well as the risk of other clinical manifestations of progressive SVD (ischaemic stroke, dementia, late-life depression and gait impairment) in a cohort of consecutive ICH survivors.

## Materials and methods

### Patient recruitment and baseline data collection

**Figure 1:**
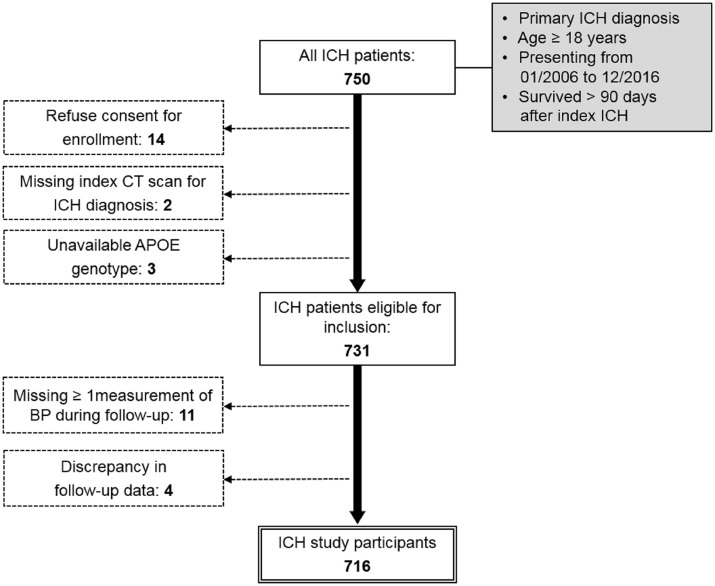
**Study inclusion/exclusion criteria and enrolment flow diagram.** Figure presents study design, inclusion/exclusion criteria and composition of patient group retained for analysis. Solid single-line boxes represent subjects meeting criteria for inclusion in the present study at each step. Eligibility criteria for inclusion in the study are listed in grey-background boxes. Dashed lines connect to dashed-bordered boxes listing criteria for exclusion and the number of subjects excluded. The double-line bordered box indicates the final study group selected for analyses mentioned in the Results section. ICH = intracerebral haemorrhage.

All participants were enrolled in an ongoing single-centre longitudinal cohort study of ICH as previously described (Biffi *et al.*, 2010*a*; Biffi *et al.*, 2012; Biffi *et al.*, 2015), and selected based on the following inclusion criteria: (i) age ≥18 years; (ii) admitted to Massachusetts General Hospital from January 2006 to December 2016; (iii) diagnosed with spontaneous ICH confirmed by CT scan (Fig. 1); and (iv) survived at least 90 days after index ICH (Biffi *et al.*, 2015). *APOE* genotypes for variants ɛ2 and ɛ4 were determined from DNA samples derived from peripheral venous blood, drawn at time of enrolment (Biffi *et al.*, 2010*b*; Biffi *et al.*, 2011). The study protocol was approved by the Massachusetts General Hospital Institutional Review Board. Written informed consent was obtained from all study participants or their surrogates. Additional information on recruitment and data collection can be found in the Supplementary Appendix.

### Longitudinal follow-up

ICH survivors and/or their caregivers were contacted and interviewed by dedicated study staff at 3, 6, 12 months after index ICH, and every 6 months thereafter, based on established protocols (Biffi *et al.*, 2015). Investigators obtained information about ICH recurrence, ischaemic stroke (and subtype based on the TOAST method; Adams *et al.*, 1993), cognitive impairment, gait impairment, mood impairment, death and medication use/dosing. Cognitive performance was evaluated using the Modified Telephone Interview for Cognitive Status (TICS-m) test and the 16-item (short) version of the Informant Questionnaire on Cognitive Decline in the Elderly (IQCODE-16; Brandt *et al.*, 1988; Jorm, 1994; de Jager *et al.*, 2003; Barber and Stott, 2004; Knopman *et al.*, 2010; Seo *et al.*, 2011; Pendlebury *et al.*, 2013). Incident depression was identified using the four-item version of the Geriatric Depression Scale (GDS-4; Almeida and Almeida, 1999; Pocklington *et al.*, 2016). Gait impairment was defined as a newly developed requirement for assistance (caregiver or device) for everyday ambulation, as determined by the patient and/or caregiver report. Additional information on follow-up methodology can be found in the Supplementary Appendix. At each follow-up, time-point study staff also captured information on BP measurements as previously described (Biffi *et al.*, 2015). In brief, study staff inquired about the most recent BP measurements obtained in a medical setting by medical personnel. If participants could not provide reliable BP measurements, medical records were obtained for review. We pre-specified data capture targets of ≥1 BP measurement per 6-month period.

### Variables’ definitions

*APOE* genotype was represented by two dichotomous variables indicating presence versus absence of at least one copy of ɛ2 or ɛ4. We defined incident dementia for outcome analyses as meeting at least one of these criteria: (i) subjects assigned TICS-m scores <20 (Barber and Stott, 2004; Pendlebury *et al.*, 2013); and (ii) subjects assigned average IQCODE-16 score >3.3 (Harrison *et al.*, 2014, 2015). We defined incident depression as GDS-4 score >2 (Almeida and Almeida, 1999). Gait impairment was defined as described above. To assess the role of BP in ICH recurrence we initially analysed two time-varying variables: (i) SBP as a continuous variable; and (ii) DBP as a continuous variable (Biffi *et al.*, 2015). We also analysed the following hypertension categories based on the 2017 American College of Cardiology/American Heart Association (ACC/AHA) high BP guidelines (Whelton *et al.*, 2018): (i) normal BP (SBP <120 mmHg and DBP <80 mmHg); (ii) elevated BP (SBP 120–129 mmHg and DBP <80 mmHg); (iii) hypertension stage 1 (SBP 130–139 mmHg or DBP 80–90 mmHg); (iv) hypertension stage 2 (SBP ≥ 140 mmHg or DBP ≥ 90 mmHg).

### Statistical analysis

Separate statistical models were created for each outcome of interest, as well as for a composite outcome including all SVD-related clinical diagnoses (recurrent ICH, ischaemic stroke, dementia, depression and gait impairment). We determined factors associated with each outcome using log-rank tests (univariable analyses) and Cox regression (multivariable analyses). Additional details on multivariable modelling are provided in the Supplementary Appendix. We conducted interaction analyses for SBP/DBP with *APOE* ɛ2/ɛ4 if primary terms were found to be significant in multivariable analyses. We then separately performed analyses stratifying subjects by both hypertension severity (based on the 2017 ACC/AHA guidelines) and *APOE* genotype. We estimated yearly risk for SVD outcomes of interest for graphical plotting purposes, by combining the Nelson-Aalen cumulative hazard function with the Cox model determined statistical risk effects using the *predictSurvProb* function in the *pec* R package. Estimated risks were graphically subdivided based on: (i) number of *APOE* ɛ4 copies and (ii) hypertension severity (per 2017 ACC/AHA guidelines) during follow-up. We found that SVD outcomes showed significant correlation with each other (Supplementary Table 1), and therefore, did not meet the criteria for Bonferroni adjustment. We, therefore, addressed multiple testing burden by adopting the false discovery rate method (Keselman *et al.*, 2002.). All significance tests were two-tailed, and the significance threshold was set at *P* < 0.05 (after false discovery rate adjustment). All analyses were performed with R software v 3.5.2 (The R Foundation for Statistical Computing). Additional information on the statistical methodology can be found in the Supplementary Appendix.

### Literature review and attempted replication of results

To attempt external replication of our findings, we conducted a search of published literature and publicly available data, to identify suitable datasets for analysis. We searched PubMed, Embase, Ovid, Google Scholar, Dryad, Figshare, Zenodo and OSF for articles and data published prior to August 2018, using a dedicated electronic search strategy (see details in Supplementary Appendix). We selected for further manual review studies that (i) included only patients diagnosed with primary (i.e. spontaneous) ICH; and (ii) studies that had either APOE genotype or BP data available in the original dataset.

### Data availability

The data that support the findings of this study are available from the corresponding author upon reasonable request.

## Results

### Study participants

A total of 750 patients met the initial criteria for inclusion (Fig. 1). Patients who declined consent (*n* = 14) were missing an index CT scan (*n* = 2) or had no available *APOE* genotype (*n* = 3) were excluded from all analyses. A total of 11 participants were missing BP measurements for one or more 6-month periods (Fig. 1), and were removed from all analyses; their forced re-introduction did not alter results substantially (Supplementary Table 2). Discrepancies between telephone-collected and EMR-collected follow-up data resulted in the removal of four patients from the present study (Fig. 1), and their removal did not alter results substantially (Supplementary Table 3). A total of 716 patients (Table 1) were, therefore, included in our analyses.

**Table 1:** Participants’ characteristics

Variable	No. of subjects	%
	716	100
Demographics		
Age (mean, SD)	70.5 (12.2)	
Sex (male)	385	53.8
Race/ethnicity		
European American	599	83.7
African American	47	6.6
Asian American	27	3.8
Hispanic	38	5.3
Other	5	0.7
Education (≥10 years)	418	58.4
ICH location		
Lobar	323	45.1
Non-lobar	393	54.9
Pre-ICH medical history		
Hypertension	531	74.2
Ischaemic heart disease	129	18.0
Atrial fibrillation	131	18.3
Diabetes	138	19.3
Pre-ICH dementia	64	8.9
Pre-ICH mood disorder	99	13.8
Pre-ICH gait impairment	34	4.8
Prior ICH (before index event)	37	5.2
Prior ischaemic stroke/TIA	85	11.9
*APOE* genotype		
* APOE ɛ2* (frequency)	0.09	
*APOE ɛ4* (frequency)	0.19	
Post-ICH medication use		
Antiplatelet agents	115	16.0
Warfarin	75	10.5
Statins	278	38.8
Antihypertensive agents	509	71.0
SSRI	236	32.9

ICH = intracerebral haemorrhage; SSRI = selective serotonin reuptake inhibitors.

### Follow-up information and post-ICH outcome rates

During a median follow-up time of 52.8 months [inter-quartile range (IQR) 29.8–69.5), we observed an average rate of loss to follow-up of 1.4% per year. We observed a total of 89 recurrent ICH events among 716 study participants, corresponding to a recurrence rate of 3.4%/year (95% CI 2.1–5.4%), and 59 ischaemic stroke events, corresponding to an incidence rate of 2.1%/year (95% CI 1.4–3.0%). Among ischaemic stroke events, 21 (36%) were categorized as small vessel stroke by TOAST criteria, corresponding to an incidence rate of 0.9%/year (95% CI 0.3–1.4%). A total of 122/716 (17%) ICH survivors developed new-onset dementia, corresponding to an incidence rate of 5.2%/year (95% CI 4.6–5.7%). We observed that 182/716 (25%) ICH survivors developed new-onset depression, corresponding to an incidence rate of 6.4%/year (95% CI 5.7–6.9%). Finally, 95/716 (13%) participants developed new-onset gait impairment, corresponding to an estimated incidence rate of 3.6%/year (95% CI 3.1–4.0%). We present detailed information on study sample size, mortality, loss to follow-up and post-ICH outcomes during the first 5 years of follow-up in Supplementary Table 4.

### *APOE* genotype, hypertension and outcome after intracerebral haemorrhage

In univariable analyses (Supplementary Table 5), SBP and *APOE* ɛ4 were associated with risk of recurrent ICH, small vessel ischaemic stroke, incident dementia, incident depression and incident gait impairment. These findings were confirmed in multivariable analyses (Table 2), after adjustment for relevant covariates (see Supplementary Appendix for additional details).

**Table 2:** Multivariable analyses of association between *APOE*, BP and post-ICH outcomes

Risk factors^a^	Post-ICH outcomes									
	ICH recurrence		Small vessel ischaemic stroke		Incident dementia		Incident depression		Gait impairment	
	HR (95% CI)	*P*	HR (95% CI)	*P*	HR (95% CI)	*P*	HR (95% CI)	*P*	HR (95% CI)	*P*
*APOE* genotype										
*APOE* ε2(≥ 1 copy)	1.26 (0.56—2.83)	0.58	1.06 (0.84–1.33)	0.62	1.32 (0.79–2.20)	0.29	0.89 (0.45–1.78)	0.74	1.21 (0.80–1.83)	0.37
*APOE* ε4(≥ 1 copy)	**1**.**87 (1**.**20–2**.**92)**	**0.006**	**1**.**19 (1**.**01–1**.**41)**	**0**.**047**	**1**.**85 (1**.**21–1**.**84)**	**0**.**005**	**1**.**70 (1**.**10–2**.**63)**	**0**.**018**	**1**.**56 (1**.**12–2**.**18)**	**0**.**01**
*BP measures*										
SBP (10 mmHg increase)	**1**.**33 (1**.**06–1**.**66)**	**0**.**012**	**1**.**25 (1**.**01–1**.**57)**	**0**.**039**	**1**.**69 (1**.**14–2**.**50)**	**0**.**009**	**1**.**23 (1**.**01–1**.**50)**	**0**.**049**	**1**.**45 (1**.**14–1**.**85)**	**0**.**003**
DBP (10 mmHg increase)	1.09 (0.99–1.20)	0.080	1.20 (0.87–1.65)	0.26	0.96 (0.88–1.04)	0.33	1.29 (0.59–2.83)	0.53	1.03 (0.92–1.15)	0.60

a All models included the following covariates for adjustment: self-reported race/ethnicity, history of prior ICH (lobar and/or non-lobar), educational level, ICH location, antiplatelet agent use and warfarin use.

DBP = diastolic blood pressure; ICH = intracerebral haemorrhage; SBP = systolic blood pressure.

We subsequently tested for interaction between SBP and *APOE* ɛ4 in multivariable analyses, and found association with increased risk of recurrent ICH, small vessel ischaemic stroke, incident dementia and incident gait impairment (Supplementary Table 6, interaction *P* < 0.05 for ICH recurrence, small vessel ischaemic stroke, dementia and gait impairment). We repeated multivariable analyses for dementia, depression and gait impairment for subjects who did not experience ICH or ischaemic stroke during follow-up (*n* = 566). In this subset, we identified consistent interaction between SBP and *APOE* ɛ4 in determining the risk of dementia and gait impairment (Supplementary Table 7).

**Figure 2:**
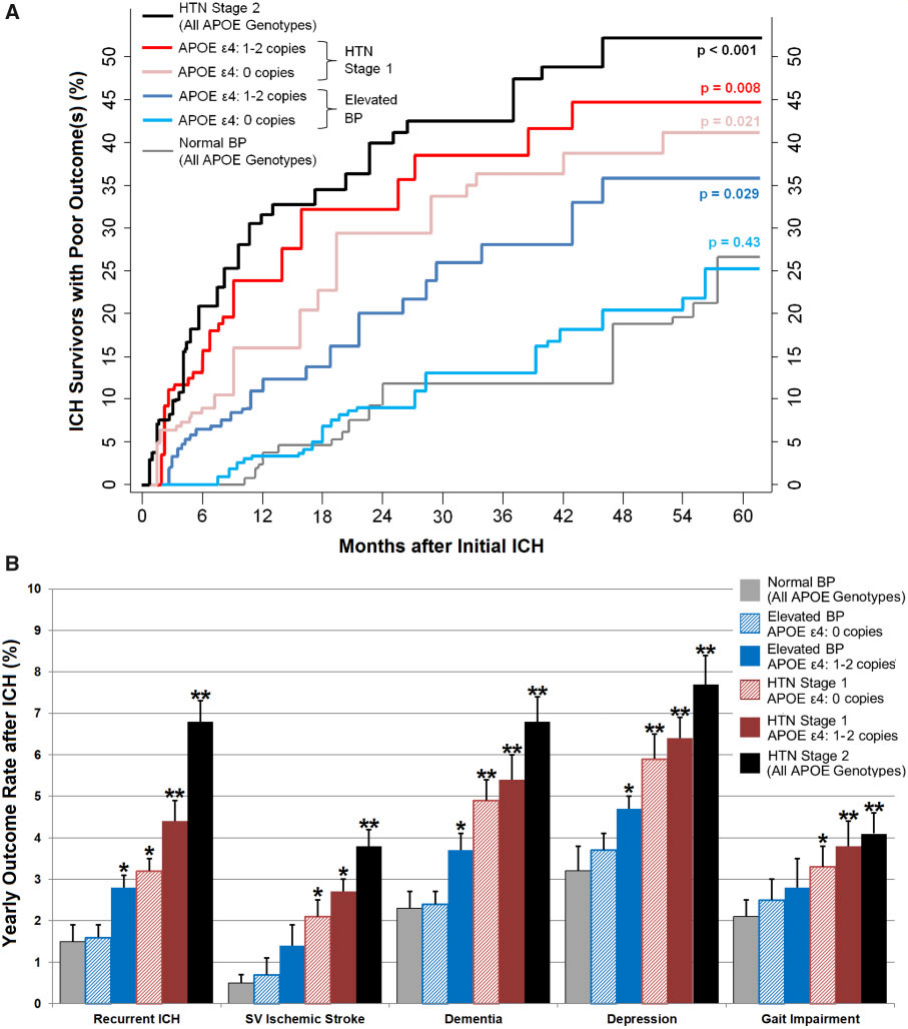
**Risk of poor outcomes after ICH, based on *APOE* genotype and hypertension severity during follow-up.** (**A**) Kaplan–Meier estimates of risk for composite poor post-ICH outcome, including: (i) recurrent ICH; (ii) ischaemic stroke (all subtypes); (iii) incident dementia; (iv) incident depression; and (v) incident gait impairment. Risk distributions are separated based on *APOE* genotype (ε4: 0 copies versus 1–2 copies) and hypertension severity during follow-up. *P*-values are calculated for each group in reference to normotensive subjects (regardless of *APOE* genotype) using the Log-rank test. (**B**) Estimates of yearly risk for individual post-ICH outcomes of interest, stratified by *APOE* genotype (ε4: 0 copies versus 1–2 copies) and hypertension severity during follow-up. Vertical error bars indicate one standard deviation in risk estimate. Single asterisk indicates *P*-value < 0.05 for comparison with normotensive subjects (regardless of *APOE* genotype) using the Log-rank test. Double asterisks indicate *P*-value < 0.01 for comparison with normotensive subjects (regardless of *APOE* genotype) using the Log-rank test. ICH = intracerebral haemorrhage; SV = small vessel.

To quantify *APOE*-dependent effects on the relationship between BP and post-ICH outcomes, we first explored associations between *APOE* ɛ4 and the composite post-ICH poor outcome endpoint (including recurrent ICH, small vessel ischaemic stroke, dementia, depression and gait impairment) within each hypertension severity category. We found that *APOE* ɛ4 was associated with increased risk for poor outcome among patients with elevated BP, hypertension stages 1 and 2 (Supplementary Table 8). We then repeated all multivariable analyses after stratification for hypertension severity (according to 2017 ACC/AHA guidelines) and number of ɛ4 copies (Table 3). These analyses demonstrated that non-hypertensive ICH survivors with elevated BP (i.e. SBP of 120–129 mmHg and DBP <80 mmHg) were at increased risk for a composite endpoint of recurrent ICH, small vessel ischaemic stroke, dementia, depression and gait impairment only if they possessed one or more *APOE* ɛ4 copies: hazard ratio (HR) = 1.97, 95% confidence interval (CI) 1.17–3.31, *P* = 0.011 for comparison between participants with elevated BP with versus without APOE *ɛ4* (Fig. 2A). Specifically, *APOE ɛ4* carriers with elevated BP showed significant differences in risks for recurrent ICH (HR = 2.11, 95% CI 1.06–4.21, *P* = 0.036), dementia (HR = 1.89, 95% CI 1.05–3.41, *P* = 0.037) and depression (HR = 1.66, 95% CI 1.02–2.70, *P* = 0.044) as detailed in Fig. 2B. ICH survivors with hypertension Stage 1 and beyond were also at increased risk for poor outcomes after ICH, with *APOE* genotype further increasing risk among those with one or more ɛ4 copies (Fig. 2A and B). We provide detailed results of association analyses for the composite post-ICH poor outcome endpoint (including recurrent ICH, small vessel ischaemic stroke, dementia, depression and gait impairment), stratified by APOE genotype and hypertension severity, in Supplementary Table 9. Finally, we estimated whether modelling the SBP/*APOE ɛ4* genotype interaction improved predictive ability for post-ICH outcomes, and found significant differences (compared with models including both variables but no interaction term) for prediction of future risk of ICH recurrence, ischaemic stroke, dementia, depression and gait impairment (Supplementary Table 10).

**Table 3:** Hypertension severity and post-ICH outcomes, stratified by *APOE ε4* genotype

Risk factors^a^	Post-ICH outcomes									
	ICH recurrence		Small vessel stroke		Incident dementia		Incident depression		Gait impairment	
	HR (95% CI)	*P*	HR (95% CI)	*P*	HR (95% CI)	*P*	HR (95% CI)	*P*	HR (95% CI)	*P*
*APOE* ε4: 0 copies										
Normal BP	Ref.	Ref.	Ref.	Ref.	Ref.	Ref.	Ref.	Ref.	Ref.	Ref.
Elevated BP	1.68 (0.54–5.20)	0.19	1.39 (0.92–2.10)	0.12	1.23 (0.60–2.50)	0.57	1.36 (0.98–1.88)	0.07	1.12 (0.95–1.32)	0.20
Hypertension: Stage 1	**2.21 (1.14–4.30)**	**0.020**	**1.88 (1.02–3.46)**	**0.044**	**2.14 (1.25–3.67)**	**0.006**	**1.54 (1.10–2.16)**	**0.013**	1.81 (0.91–3.59)	0.10
Hypertension: Stage 2	**3.54 (1.40–8.92)**	**0.004**	**2.19 (1.07–4.47)**	**0.033**	**2.58 (1.23–5.42)**	**0.013**	**2.39 (1.26–4.53)**	**0.009**	**2.35 (1.19–4.46)**	**0.015**
*APOE* ε4: ≥1 copy										
Normal BP	Ref.	Ref.	Ref.	Ref.	Ref.	Ref.	Ref.	Ref.	Ref.	Ref.
Elevated BP	**2.11 (1.23–3.63)**	**0.004**	**1.69 (1.08–2.64)**	**0.022**	**1.90 (1.22–2.95)**	**0.004**	1.25 (0.88–1.78)	0.22	**1.74 (1.21–1.50)**	**0.003**
Hypertension: Stage 1	**3.05 (1.14–6.46)**	**0.002**	**2.49 (1.25–4.95)**	**0.01**	**2.81 (1.27–6.20)**	**0.011**	**1.60 (1.15–2.23)**	**0.007**	**2.84 (1.20–6.71)**	**0.018**
Hypertension: Stage 2	**4.21 (1.53–11.59)**	**0.003**	**3.12 (1.41–6.92)**	**0.006**	**4.08 (1.58–10.41)**	**0.004**	**2.77 (1.23–6.21)**	**0.015**	**3.33 (1.34–8.25)**	**0.01**

a All models included the following covariates for adjustment: self-reported race/ethnicity, history of prior ICH, educational level, ICH location, antiplatelet agent use and warfarin use.

ICH = intracerebral haemorrhage; BP = blood pressure.

### Systematic review and attempted replication of results

After reviewing published literature and publicly available data, we identified 50 original reports of ICH survivors (see Supplementary Appendix). Of these, 16 were conducted at Massachusetts General Hospital and enrolled participants also included in the present study. Of the remaining 34 studies, 16 included *APOE* genotype for enrolled ICH survivors and only 2 had available BP data during follow-up. Of the latter, one included *APOE* genotype but only evaluated ICH recurrence as the outcome of interest. We, therefore, concluded that independent replication of our findings was not feasible at the time of our analyses.

## Discussion

We demonstrate that *APOE* ɛ4 interacts with BP following primary ICH to increase the risk for recurrent ICH, small vessel ischaemic stroke, incident dementia and incident gait impairment. This effect extended to non-hypertensive ICH survivors with SBP of 120–129 mmHg and DBP <80 mmHg) who were at increased risk for a composite endpoint of recurrent ICH, small vessel ischaemic stroke, dementia, depression and gait impairment only if they possessed *APOE* ɛ4. Thus, *APOE* genotype identified high-risk individuals who would otherwise be deemed to be relatively low risk. These results, which represent a unique example of interaction between a common genetic variant and a modifiable risk factor (BP) to influence multiple outcomes for a prevalent, highly relevant neurological condition.

The finding that *APOE ɛ4* modifies the association of BP with multiple common clinical manifestations of SVD among ICH survivors has clinical implications. First and foremost, counselling of ICH patients and their caregivers may benefit from the inclusion of *APOE* genotype. Carriers of *ɛ4* might be selected for closer BP monitoring and/or more aggressive management. From a broader perspective, published guidelines for BP reduction following ICH may merit reconsideration and inclusion of genetic information. We also demonstrated that modelling the interaction between *APOE ɛ4* and BP improved the predictive capability for most outcomes of interest. This is relevant for future research studies in the field of ICH and SVD; more accurate modelling of outcome risk would allow investigators to design randomized controlled trials focused on highest-risk individuals, thus maximizing success rate for identification of truly beneficial interventions (Stanley, 2007). Furthermore, clarification of the biological basis for the described interaction between *APOE* genotype and BP control may well highlight novel aspects of SVD pathophysiology, thus offering additional targets for potential intervention. Finally, the opportunity to act on a modifiable risk factor based on easily ascertained genetic data would represent an ideal ‘sandbox’ to explore psychological, economic and societal implications of potential upcoming advances in precision medicine (Gray *et al.*, 2014; Collins and Varmus, 2015; Lander, 2015).

*APOE* genotype plays a crucial role in determining the risk and severity of cerebral amyloid angiopathy (CAA), a common form of cerebral SVD characterized by accumulation of amyloid-β (Aβ) in the CNS leptomeningeal medium and small arteries (Rannikmae *et al.*, 2013). Common clinical manifestations of CAA include ICH, lacunar ischaemic stroke, cognitive and gait impairment (Biffi and Greenberg, 2011). As previously described for parenchymal Aβ accumulation, hypertension severity likely leads to worsening damage to CAA-prone arterial vessels among *APOE* ɛ4 carriers, increasing the risk for a variety of associated clinical outcomes (de Leeuw *et al.*, 2004; de Frias *et al.*, 2014; Kester *et al.*, 2014). Due to the frequent co-existence of vascular Aβ pathology (in the form of CAA) and parenchymal Aβ pathology (in the form of Alzheimer’s disease), a proportion of the interaction effects we identified likely reflects known relationships between hypertension severity and Alzheimer’s disease progression (Smith and Greenberg, 2009). Taken together, these considerations reflect the established role of *APOE* in risk for Alzheimer’s disease/CAA by dint of their role in Aβ aggregation, deposition and clearance (Kanekiyo *et al.*, 2014). However, *APOE* gene products also play a critical role in non-amyloid biological pathways, including inflammatory response (Tai *et al.*, 2015), CNS lipid homeostasis (Mahley, 2016), neurogenesis and synaptic plasticity (Liu *et al.*, 2013; Kim *et al.*, 2014) and mitochondrial resistance to oxidative stress (Jofre-Monseny *et al.*, 2008). As a result, *APOE* ɛ4 acts directly or in concert with age, head injury, oxidative stress, ischaemia and inflammation to alter disease onset, progression and prognosis in a variety of neurological disorders (Maiti *et al.*, 2015). Finally, *APOE* ɛ4 has also been directly linked with cerebrovascular dysfunction via a variety of mechanisms, including pericyte migration/activation, astrocyte activation, smooth muscle cell damage, basement membrane degradation and alterations in brain endothelial cells (Zlokovic, 2013; Tai *et al.*, 2016). We, therefore, hypothesize our findings to reflect more broadly the biological interaction between the damaging effects of hypertension and the pathological substrate represented by *APOE* ɛ4 across a multitude of mechanistic pathways.

The robustness of our results is supported by the strengths of our design, which include consistent capture and incorporation of relevant neuroimaging, genetic and BP information and follow-up based on standardized procedures, capturing multiple relevant outcome endpoints of immediate clinical relevance. The study’s limitations derive most substantially from its small sample size. Furthermore, subjects were recruited and followed in an observational manner at a single academic tertiary care centre. These findings will, therefore, require replication in different centres and healthcare delivery settings. In our systematic review, we were unable to identify currently available resources that would allow for a ready replication of our findings. In addition, we were limited in our characterization of cognitive, mood and gait disorders. Specifically, our ability to better delineate cognitive impairment by sub-domains, capture severity of mood symptoms or describe patterns of gait impairment is minimal, due to the nature of the screening tools employed. Finally, BP data capture was non-standardized and likely resulted in imprecision in capturing hypertension severity. However, this is unlikely to have systematically affected individuals based on *APOE* genotype, and more likely eroded statistical power instead of generating false-positive associations.

In summary, we demonstrate that *APOE* genotype interacts with average SBP to influence long-term clinical outcomes following ICH. Among non-hypertensive ICH survivors with elevated BP, *APOE* genotype identified those at higher risk for poor outcome. Although these findings require replication, their incorporation in clinical practice and future clinical trials may guide precision approaches for BP control in this very high-risk population.

## Supplementary Material

fcz018_Supplementary_Materials

## Abbreviations

95% CI: 95% confidence interval
Aβ: amyloid-β
BP: blood pressure
CAA: cerebral amyloid angiopathy
DBP: diastolic blood pressure
GDS-4: 4-item version of the Geriatric Depression Scale
HR: hazard ratio
ICH: intracerebral haemorrhage
IQR: inter-quartile range
IQCODE-16: 16-item (short) version of the Informant Questionnaire on Cognitive Decline in the Elderly
SVD: small vessel disease
SBP: systolic blood pressure
TICS-m: Modified Telephone Interview for Cognitive Status
