# Supplementary material for: APOE genotype, hypertension severity and outcomes after intracerebral haemorrhage
Source: Brain Commun. 2019 Sep 14;1(1):fcz018. doi: 10.1093/braincomms/fcz018 (PMC7425529; doi:10.1093/braincomms/fcz018)
Supplement: fcz018_Supplementary_Materials [file fcz018_supplementary_materials.zip › fcz018_Supplementary_material_Appendix.pdf]

APOE Genotype, Hypertension

and Outcomes after Intracerebral Hemorrhage

Biffi A et al.

APPENDIX

INDEX

CONTENT	PAGE
**Index**	**Page S1**
**List of Abbreviations**	**Page S2**
**Supplementary Methods**	**Page S3**
- Enrollment Procedures	Page S3
- Neuroimaging Data Analyses	Page S4
- Genetic Data Analyses	Page S4
- Initial In-person Follow-up	Page S4
- Follow-up Protocol	Page S4
- Blood Pressure Data Capture	Page S5
- Outcome Variables' Definition	Page S5
- Univariable Statistical Analyses	Page S6
- Multivariable Statistical Analyses	Page S6
- Post-ICH Clinical Deterioration Risk Estimation	Page S7
- Literature and Data Search Strategy	Page S8
**Supplementary Table S1**	**Page S9**
**Supplementary Table S2**	**Page S10**
**Supplementary Table S3**	**Page S11**
**Supplementary Table S4**	**Page S12**
**Supplementary Table S5**	**Page S13**
**Supplementary Table S6**	**Page S14**
**Supplementary Table S7**	**Page S15**
**Supplementary Table S8**	**Page S16**
**Supplementary Table S9**	**Page S17**
**Supplementary Table S10**	**Page S18**
**References**	**Page S19**

LIST OF ABBREVIATIONS:

ADLs = Activities of Daily Living
BP = Blood Pressure
CT = Computerized Tomography
CCS = Causaitive Classification of Stroke
DBP = Diastolic Blood Pressure
GDS-4 = 4-item version of the Geriatric Depression Scale
HR = Hazard Ratio
IADLs = Instrumental Activities of Daily Living
ICH = Intra-Cerebral Hemorrhage
IQCODE = Informant Questionnaire on Cognitive Decline in the Elderly
IQR = Inter-Quartile Range
MA = Certified Medical Assistant
MD = Doctor of Medicine
mRS = modified Rankin Scale
NIH = National Institutes of Health
NIHSS = National Institutes of Health Stroke Scale
NP = Licensed Nurse Practitioner
OMB = Office for Management and Budget
OT = Occupational Therapy
PA = Certified Physician Assistant
PH = Proportional Hazards
PT = Physical Therapy
RN = Registered Nurse
SBP = Sytolic Blood Pressure
SLP = Speech and Language Pathology
TIA = Transient Ischemic Attack
TICS = Telephone Interview for Cognitive Status
VIF = Variance Inflation Factor

SUPPLEMENTARY METHODS

Enrollment Procedures

ICH patients or their guardians / surrogate decision makers were contacted for consideration of enrollment in our longitudinal ICH follow-up cohort within 24 hours of admission with new diagnosis of primary ICH. All participants in the present study were thus enrolled within 24 hours of presentation. Trained study staff obtained written informed consent for participation in this study (including for genetic testing via peripheral venous blood draw) from all patients or guardians / surrogate decision makers.

An enrollment in-person interview and review of medical records were then conducted to capture detailed information on:

- Demographics:
  - Age: self-reported (confirmed by study staff with medical records, identification information)
  - Sex: self-reported
  - Race/Ethnicity: self-reported from categories recommended by the OMB for all NIH-funded clinical research with patients (http://www.whitehouse.gov/omb/fedreg_1997standards).
  - Education: self-reported (in number of years of formal schooling)

- Past medical history: self-reported (cross-checked by study staff with admission information and electronic medical records for patients already receiving care within our academic hospital network).
  Specific conditions directly enquired about regardless of self-report included:
  - Prior ICH (before current event), confirmed via review of imaging and categorized based on location (lobar, deep, cerebellar, multiple locations - see below).
  - Prior Ischemic Stroke, confirmed via review of imaging, etiological subtyping performed from available medical records using the CCS system
  - Prior TIA (based on review of medical records)
  - Prior Dementia: At time of enrollment in our study reliable informants were identified for each participant, and were administered the 16-item (short) version of the IQCODE. As per reported normative data, any subjects with an average score (across all items) of > 3.3 was deemed to have suffered from pre-ICH dementia.[1,2]
  - Prior Depression: based on self-report, informant interview and review of medical records
  - Prior Gait Impairment: defined as device or caregiver assistance for ambulation on flat surfaces, as inferred from self-report, informant interview and review of medical records
  - Ischemic Heart Disease (defined as history of angina and/or acute MI)
  - Diabetes mellitus (per American Diabetes Association diagnostic criteria)
  - Hypertension (defined as ≥ 1 BP measurement with SBP > 140 mmHg OR SBP > 90 mmHg)
  - Dyslipidemia (any type of serum lipid fraction abnormality)
  - Atrial fibrillation (documented by medical records or cardiac electrophysiological data)

- Pre-ICH medication exposures: self-reported (cross-checked by study staff with admission information and electronic medical records for patients already receiving care within our academic hospital network).

- Pre-ICH functional status:
  - via structured questionnaire to determine pre-ICH mRS
  - via structured questionnaire to identify dependence for ADLs
  - via structured questionnaire to identify dependence for IADLs

- Acute ICH clinical presentation information:
  - Time from symptoms' onset to ED arrival: obtained from ED medical records

- Presentation NIHSS: obtained from admitting neurology attending physician notes
- Information on admission (within 24 hours) CT availability

In order to be considered eligible for further follow-up patient required (at a minimum):

- CT-confirmed diagnosis of primary ICH (including anatomical location, see below)
- complete demographic information (as above)
- past medical history data (reconciled information from self-report and medical records)
- pre-ICH medication exposure data
- pre-ICH mRS
- admission NIHSS
- APOE genotype determined from peripheral blood sample (see below)

Neuroimaging Data Analyses

CT Scans were evaluated by study staff (stroke neurologist and/or neuroradiologists) blinded to participants' clinical and genetic information. ICH location was assigned to one of the following categories: lobar (selective involvement of cerebral cortex and underlying white matter), deep (selective involvement of thalami and/or basal ganglia and/or brainstem), cerebellum (selective unilateral or bilateral cerebellar involvement), or multiple locations. Additional ICH location information included laterality and anatomical cortical lobes involved (frontal, parietal, temporal, occipital).

Genetic Data Analysis

All DNA samples were isolated from fresh or frozen peripheral venous blood within 6 hours of blood draw, quantified using a Quantification Kit, and normalized to a concentration of 30 ng/ul. Two genotype-determining variants in APOE, rs7412 and rs429358, were independently genotyped using two separate assays. The allelic reads from the two assays were then translated to APOE genotypes (ε3ε3, ε3ε4, ε4ε4, ε3ε2, ε2ε2 and ε2ε4). All patient groups (regardless of BP measurements / categories) were found to be in Hardy-Weinberg equilibrium for APOE genotypes.

Initial In-person Follow-up

All study participants who survived > 90 days following ICH were re-evaluated in person at ~ 3 months after ICH, in the setting of a clinical visit including general physical and neurological examination (including cognitive testing where appropriate), BP measurement (including both SBP and DBP), laboratory studies as deemed necessary by the attending physician and (where applicable) additional neuroimaging. Based on all available information the attending stroke neurologist confirmed a diagnosis of primary ICH.

Follow-up Protocol

Patients were contacted via phone interview at 6, 9, 12 months after index ICH, and every 6 months thereafter by certified study staff having undergone dedicated training. During the follow-up phone calls, study staff inquired about recurrent ICH events (if otherwise not identified), incident ischemic stroke, incident dementia, incident depression, incident gait impairment or death if contacting guardian / legal proxy for deceased patient.

In case of recurrent ICH or incident ischemic stroke, additional medical records (including neuroimaging reports and raw data on disk) were obtained for confirmation and further characterization, using identical methods to those listed above (see **Enrollment Procedures**).

In case of death, relevant information (including radiographic reports and/or autopsy reports) was obtained about cause of death from guardian / legal proxy. We also queried the Social Security Death Index (SSDI) national database as an alternative way of identifying deaths among ICH survivors. SSDI querying is performed by trained study staff on all patients currently enrolled every 3-6 months. Identification of a non-recorded death event via SSDI triggered a one-time follow-up phone call (regardless of follow-up schedule) for confirmation of patient death and ascertainment of cause of death.

Relevant records were also retrieved, either via EMR review or from former guardians / care-givers of deceased ICH patients contacted by phone.

Study staff also obtained the following information:

- updated medication exposure data (cross-checked by study staff with electronic medical records and pharmacy records with patients' consent)
- global cognitive functioning (using the TICS questionnaire)
- screening for incident depression (using the GDS-4 scale)
- ability to walk on flat surfaces without needing assistance from either device or caregiver (information obtain from self-report / informant and cross-checked with medical records; for the purpose of this evaluation, assistive devices included one or more canes, walker or hemi-walker, and wheelchair, motorized or otherwise).
- dependence for ADLs (via structured questionnaire)
- dependence for IADLs (via structured questionnaire)

Since we sought to investigate multiple outcomes, we censored subjects from the study in its entirety only in case of death or loss to follow-up. Loss to follow-up was defined as meeting one or more of the following criteria: 1) death with inability to document non-ICH cause of death; 2) documented geographical relocation with inability to re-establish phone contact; 3) documented disconnection of telephone line provided for follow-up phone calls with inability to re-establish phone contact; 4) otherwise unable to connect via phone with participant or informant within 180 days of scheduled date for follow-up call. Subjects were also censored if they failed to meet pre-specified criteria for BP measurement availability, i.e. ≥ 1 measurements (including SBP AND DBP per 6 months' interval, see also below).

Blood Pressure Data Capture
During all follow-up phone calls study staff inquired about measured SBP and DBP values.
In order to qualify for recording, recalled BP measurements had to be:

- Obtained by a medical provider (MD, MA, PA, NP, PT/OT/SLP certified therapist or RN)
- Obtained in a medical setting: inpatient hospital facility, rehabilitation facility, emergency department, urgent care clinic, primary care clinic, or outpatient specialist clinic
- Obtained since prior telephone follow-up call
- Consist of precisely recalled SBP AND DBP

If subjects could recall BP measurements being taken - but not their exact value - two additional strategies for data capture were employed:

- For BP measurements reportedly obtained at our institution or affiliated medical facility, EMR was queried to retrieve medical data
- For BP measurements reportedly obtained outside our institution, patients were asked to retrieve relevant medical records and provide them via phone (next follow-up phone call), fax or mail at patients' discretion.

A total of 17,293 SBP/DBP measurements were recorded and available for analysis in the present study. A total of 580 of 716 study participants (81%) had 100% of BP measurements recorded in the MGH EMR system or other medical records, for a total of 12,278 out of 17,293 BP measurements (71%). There were no study participants with 100% BP measurements obtained from telephone-based self-report. The highest percentage of self-reported BP measurements was 3 out of 9 (33%), while the highest self-reported BP measurements count was 7 out of 25 (28%).
Among 12,278 BP measurements obtained from medical records, a total of 7,129 (41% of all study BP measurements) had simultaneously obtained values from patient self-report over the phone. Among these BP measurements, we noted good correlation between medical records and patient self-report for both SBP (Spearman's correlation coefficient 0.84, $p < 0.001$) and DBP (Spearman's correlation coefficient 0.79, $p < 0.001$). Median absolute SBP difference in mmHg between medical records and self-report value was 3 mmHg (IQR 0 – 7). Median absolute DBP difference in mmHg between medical records and self-report value was 2 mmHg (IQR 0 – 6).

Outcome Variables' Definition

Recurrent ICH was defined as a novel, imaging- or autopsy- confirmed intracerebral bleeding event, deemed by the attending stroke neurologist on the study staff to represent a repeat primary ICH event. Incident ischemic stroke was defined as new-onset, ictal neurological signs and symptoms attributable to CNS ischemia based on the attending stroke neurologist's evaluation, and confirmed to represent such by evidence of diffusion-weighted signal abnormalities on MRI Brain obtain within 24 hours of symptoms' onset. For all documented ischemic stroke etiological subtyping was performed using the TOAST system, to identify small vessel stroke events (i.e. lacunar infarcts).

We defined incident dementia as participants meeting at least one of these criteria on least one time-point during follow-up: 1) subjects assigned TICS-m scores < 20 based on previously reported normative data;[3] 3) subjects assigned average score > 3.3 on short version of the IQCODE.[1,2] At total of 358 of 716 study participants (50%) had a diagnosis of incident dementia formally specified as present or absent by a board-certified neurologist in a documented clinical encounter in our EMR system. Using clinical evaluation as gold standard, our operational criteria for diagnosis of dementia had sensitivity / specificity of 93% / 95% respectively. A total of 129 of 716 study participants (18%) had a diagnosis of incident dementia formally specified as present or absent via standardized neuropsychological testing administered by a certified neuropsychologist. Using neuropsychological testing as gold standard, our operational criteria for diagnosis of dementia had sensitivity / specificity of 91% / 94% respectively.

We defined incident depression as a score > 2 on the GDS-4 based on published evidence.[4] A total of 502 of 716 (70%) study participants had a diagnosis of depressive disorder specified as present or absent by a MD (any specialty) in a documented clinical encounter in our EMR system. Using clinical evaluation as gold standard, our operational criteria for diagnosis of depression had sensitivity / specificity of 89% / 92% respectively. A total of 229 of 716 study participants (32%) had a diagnosis of new-onset depressive disorder specified as present or absent by a mental health provider (board-certified psychiatrist, board-certified neuropsychiatrist, certified therapeutic counselor, certified psychiatric NP, certified psychiatric RN). Using evaluation by a mental health provider as gold standard, our operational criteria for diagnosis of depression had sensitivity / specificity of 93% / 91% respectively. Gait impairment was defined as described above (see **Follow-up Protocol**).

Univariable Statistical Analyses

We determined significance levels for association in univariable statistical analyses by means of the Log-Rank test, with single-variable Cox models being constructed to determine univariable effect size. We conducted separate analyses for all predictors listed in Table 1, as well as for BP measurements including: 1) continuous SBP; 2) continuous DBP. Please refer to main text for variable's definition. We performed six separate statistical analyses for each independent variable, i.e. separate analyses for risks of recurrent ICH, incident all-cause stroke, incident small vessel stroke, incident dementia, incident depression and incident gait impairment. APOE genotype was captured by two separate dichotomous variables (0/1) reflecting possession of either the ε2 or ε4 allele; therefore, ε3ε3 subjects (value of 0 for both described variables) served as reference. Detailed univariable association results for SBP, DBP and APOE variables are presented in Supplementary Table 5.

Multivariable Statistical Analyses

Variables with univariable Log-rank $p < 0.20$ were retained for inclusion into initial multivariable model, into which they were entered at the same time. APOE genotype, SBP and DBP were pre-selected for inclusion in multivariable modeling (regardless of univariable p-value) on the basis our study's hypothesis. BP measurements were initially entered as continuous SBP and DBP values. Six separate multivariable models were created for recurrent ICH, incident all-cause stroke, incident small vessel stroke, incident dementia, incident depression and incident gait impairment.

Multi-collinearity was tested by computing a VIF for each variable in the initial full models. Covariates with VIF > 5.0 were selected for removal from the model; however no variable in any model fulfilled this criterion. We then applied backward elimination to remove sequentially variables with $p > 0.05$ for association with ICH recurrence risk. Again, this procedure was performed separately for each of the six models listed above. Variables were removed one at a time; association statistics were re-calculated at each step, as were VIFs to check for unmasking of latent multi-collinearity (no variable fulfilled VIF-based criteria for removal). Backward elimination was repeated iteratively until minimal models containing only variables with $p < 0.05$ for association with ICH recurrence were thus generated. Of note, APOE

genotype, SBP and DBP were marked for retention in the final model regardless of association p-value, as part of the hypothesis for the present study.

Minimal model generation resulted in the following covariates (other than APOE genotype, SBP and DBP) being retained for analyses:

- ICH recurrence: Lobar ICH prior to index event, non-lobar ICH prior to index event, education status
- All-cause Ischemic Stroke: age, atrial fibrillation, prior TIA / ischemic stroke, ischemic heart disease, education status, antiplatelet agent use
- Small Vessel Ischemic Stroke: age, history of hypertension, education status
- Dementia: age, lobar ICH prior to index event, African-American ethnicity
- Depression: age, prior TIA/ischemic stroke, lobar ICH prior to index event, non-lobar ICH prior to index event
- Gait Impairment: age, ischemic heart disease, diabetes mellitus, prior TIA/ischemic stroke, lobar ICH prior to index event, non-lobar ICH prior to index event

After generation of final models, the PH assumption was tested by via graphical checks (and Schoenfeld residuals-based tests (threshold $p < 0.05$ to identify PH assumption violation). All models were found to fulfill the PH assumption using both methods.

Post-ICH Outcome Risk Estimation

We estimate yearly risk for all post-ICH outcomes of interest (i.e. recurrent ICH, incident small vessel ischemic stroke, incident dementia, incident depression and incident gait impairment), as well as for a composite poor outcome (i.e. manifesting one or more of the outcomes of interest). Estimation of post-ICH outcome risk based on average SBP and APOE ε4 genotype was performed to provide a summary graphical representation of association testing results, and made use of the *predictSurvProb* function in the *pec* R package (R v. 3.4.3). In order to model outcome risk, patients were first subdivided in groups based on average observed SBP (i.e. hypertension severity) and APOE genotype. We then calculated risk of post-ICH clinical deterioration using the Nelson-Aalen cumulative hazard estimator function in each study sub-group defined by combinations of hypertension severity and APOE genotype. Next, we identified variables (other than SBP and APOE ε4) associated with any of the post-ICH outcome of interest (see above for selection criteria). Each variable was assigned a weight based on Cox-model derived effect size on clinical deterioration risk, as identified by an HR. For each patient, individual cumulative deterioration risk during follow-up was calculated by modifying the baseline cumulative Hazard via application of weights representing HR. As a result, each study participants was assigned a percentage risk value for post-ICH outcome during follow-up, ranging from 0 to 100%. Predicted risk was then subdivided by length of follow-up (in no. of years) to account for disparity in follow-up duration. This generated an individual-level predicted risk for post-ICH outcomes of interest, again ranging from 0 to 100%. Their distribution was then plotted in Microsoft Excel using box-plot with conventions described in the legend for Figure 2, panel B (main manuscript). In each SBP/APOE-identified group, variations in yearly outcome risk are dependent on individuals' combinations of other covariate contributing to post-ICH outcomes of interest, with subjects possessing an higher number of associated factors placing higher in the distribution.

Literature and Data Search Strategy

We searched PubMed, Dryad, figshare, Zenodo and OSF for articles and data published prior to 08/2018, using an electronic search strategy for titles and abstracts combining the MeSH term “ICH or INTRACEREBRAL HEMORRHAGE ETC.” with the other study variables including “APOE” “blood pressure” “depression” “mood” “gait” “mobility” “dementia” “longitudinal” in all possible combinations. We selected for further manual review studies that 1) included only patients diagnosed with primary (i.e. spontaneous) ICH; 2) studies that had either APOE genotype or BP data available in the original dataset. We included all studies retrived as part of our search in a separate spreadsheet file.

Supplementary Table S1. Correlations between Analyzed Post-ICH Outcomes

	ICH Recurrence	Ischemic Stroke*	Dementia	Depression	Gait Impairment
**ICH Recurrence**		0.037	0.045	0.023	0.008
**Ischemic Stroke***	0.43		0.021	0.018	0.038
**Incident Dementia**	0.44	0.58		0.006	0.024
**Incident Depression**	0.51	0.59	0.62		0.035
**Gait Impairment**	0.67	0.55	0.48	0.45	

Bottom half of table reports correlation as estimated by Cramer's V statistic, ranging from 0.0 (no correlation) to 1.0 (perfect correlation). Top half of the table reports p-value for chi-square test of independence between variable pairs ($p < 0.05$ indicates greater degree or correlation than expected by chance alone).

Abbreviations: ICH = Intracerebral Hemorrhage

* includes all ischemic stroke subtypes. Small vessel ischemic stroke correlation analyses were not performed, given inclusion in the overall ischemic stroke outcome.

Supplementary Table S2. Multivariable analyses including participants missing BP measurements for one or more 6 month periods (n = 11)

Risk Factors	Post-ICH Outcomes											
	ICH Recurrence		Ischemic Stroke		Small Vessel Ischemic Stroke		Dementia		Depression		Impairment	
	**HR (95% CI)**	**p**	**HR (95% CI)**	**p**	**HR (95% CI)**	**p**	**HR (95% CI)**	**p**	**HR (95% CI)**	**p**	**HR (95% CI)**	**p**
APOE ε2 (≥ 1 copy)	1.26 (0.56 - 2.83)	0.58	0.89 (0.54 – 1.48)	0.65	1.06 (0.84 – 1.33)	0.62	1.32 (0.79 – 2.20)	0.29	0.89 (0.45 -1.78)	0.74	1.21 (0.80 – 1.83)	0.37
APOE ε4 (≥ 1 copy)	**1.87 (1.20 - 2.92)**	**0.006**	1.20 (0.80 – 1.79)	0.38	**1.19 (1.01 – 1.41)**	**0.047**	**1.85 (1.21 – 1.84)**	**0.005**	**1.70 (1.10 - 2.63)**	**0.018**	**1.56 (1.12 - 2.18)**	**0.01**
SBP (10 mmHg increase)	**1.33 (1.06 - 1.66)**	**0.012**	1.20 (0.93 – 1.55)	0.16	**1.25 (1.01- 1.57)**	**0.039**	**1.69 (1.14 – 2.50)**	**0.009**	**1.23 (1.01 - 1.50)**	**0.049**	**1.45 (1.14 – 1.85)**	**0.003**
DBP (10 mmHg increase)	1.09 (0.99 – 1.20)	0.080	1.15 (0.94 – 1.41)	0.19	1.20 (0.87 – 1.65)	0.26	0.96 (0.88 – 1.04)	0.33	1.29 (0.59 – 2.83)	0.53	1.03 (0.92 – 1.15)	0.60
Interaction - APOE ε4 - SBP	**1.65 (1.15 - 3.44)**	**0.014**	0.69 (0.21 – 2.30)	0.55	**1.32 (1.01 – 1.73)**	**0.046**	**1.48 (1.05 – 2.09)**	**0.028**	1.80 0.95 – 3.42	0.074	**1.96 (1.22 - 3.14)**	**0.006**

Abbreviations: ICH = Intracerebral Hemorrhage, SBP = Systolic Blood Pressure

Supplementary Table S3. Multivariable analyses including participants with discrepancies between telephone-collected and EMR-collected follow-up data (n = 4)

**Risk Factors**	**Post-ICH Outcomes**											
	**ICH Recurrence**		**Ischemic Stroke**		**Small Vessel Ischemic Stroke**		**Dementia**		**Depression**		**Gait Impairment**	
	**HR (95% CI)**	**p**	**HR (95% CI)**	**p**	**HR (95% CI)**	**p**	**HR (95% CI)**	**p**	**HR (95% CI)**	**p**	**HR (95% CI)**	**p**
APOE ε2 (≥ 1 copy)	1.26 (0.56 - 2.83)	0.58	1.06 (0.84 – 1.33)	0.62	1.35 (0.80 – 2.24)	0.27	0.89 (0.45 - 1.78)	0.74	1.21 (0.80 – 1.83)	0.37	1.26 (0.56 - 2.83)	0.58
APOE ε4 (≥ 1 copy)	**1.87 (1.19 - 2.95)**	**0.007**	**1.19 (1.01 – 1.41)**	**0.047**	**1.89 (1.22 – 1.91)**	**0.004**	**1.70 (1.10 - 2.63)**	**0.018**	**1.56 (1.12 - 2.18)**	**0.01**	**1.90 (1.21 - 2.95)**	**0.005**
SBP (10 mmHg increase)	**1.33 (1.06 - 1.66)**	**0.012**	**1.25 (1.01- 1.57)**	**0.039**	**1.69 (1.14 – 2.50)**	**0.009**	**1.22 (1.02 - 1.47)**	**0.047**	**1.45 (1.14 – 1.85)**	**0.003**	**1.33 (1.06 - 1.66)**	**0.012**
DBP (10 mmHg increase)	1.09 (0.99 – 1.20)	0.080	1.21 (0.90 – 1.66)	0.28	0.96 (0.88 – 1.04)	0.33	1.29 (0.59 – 2.83)	0.53	1.05 (0.93 – 1.19)	0.57	1.09 (0.99 – 1.20)	0.080
Interaction - APOE ε4 - SBP	**1.64 (1.14 - 3.45)**	**0.015**	0.69 (0.21 – 2.30)	0.55	**1.32 (1.01 – 1.73)**	**0.046**	**1.45 (1.03 – 1.98)**	**0.032**	1.80 0.95 – 3.42	0.074	**1.97 (1.22 - 3.15)**	**0.006**

Abbreviations: ICH = Intracerebral Hemorrhage, DBP = Diastolic Blood Pressure, HR = Hazard Ratio, SBP = Systolic Blood Pressure

Supplementary Table S4. Follow-up Information, Outcomes and Blood Pressure Data for Participating Subjects

Follow-up Period (in Months after ICH))	Follow-up Information			Outcome Data (n)						BP Measures	
	No. of Patients Followed	No. of Deaths	No. of Patients LTFU	Recurrent ICH Events	Ischemic Strokes	Small Vessel Strokes	Dementia Cases	Depression Cases	Gait Impairment Cases	SBP Mean (95% CI)	DBP Mean (95% CI)
**0 - 3**	716	0	0	0	0	0	0	0	0	142 (114 – 165)	79 (67 – 97)
**4 - 6**	716	0	0	2	0	0	2	6	3	139 (120 – 171)	80 (66 – 97)
**7 - 9**	716	32	0	11	5	2	8	11	6	141 (121 – 169)	80 (65 – 99)
**10 - 12**	684	41	0	17	10	5	11	17	11	139 (118 – 167)	79 (66 – 97)
**13 - 18**	643	36	0	18	9	4	14	15	10	140 (112 – 170)	78 (68 – 93)
**19 - 24**	607	26	0	9	8	2	8	12	9	141 (119 – 173)	79 (66 – 94)
**25 - 30**	581	21	0	6	5	2	12	15	11	142 (111 – 167)	78 (65 – 92)
**31 - 36**	547	28	6	4	2	0	13	16	7	140 (114 – 168)	80 (66 – 96)
**37 - 42**	514	23	10	3	4	0	12	14	8	139 (114 – 166)	80 (65 – 95)
**43 - 48**	480	22	12	5	3	2	10	11	6	137 (110 – 165)	79 (66 – 96)
**49 - 54**	451	18	11	3	2	1	7	10	6	140 (115 – 168)	77 (66 – 95)
**55 - 60**	421	21	9	2	2	1	6	16	5	141 (115 – 169)	80 (67 – 99)

Abbreviations: ICH = Intracerebral Hemorrhage, DBP = Diastolic Blood Pressure, LTFU = Loss to Follow-UP, SBP = Systolic Blood Pressure

Supplementary Table S5. Univariable analyses of association between SBP/DBP and APOE genotype with post-ICH outcomes.

**Risk Factors**	**Post-ICH Outcomes**					
	**ICH Recurrence**	**Ischemic Stroke**	**Small Vessel Ischemic Stroke**	**Dementia**	**Depression**	**Impairment**
	Log-Rank p-value	***Log-Rank p-value***	***Log-Rank p-value***	***Log-Rank p-value***	***Log-Rank p-value***	***Log-Rank p-value***
APOE ε2 (≥ 1 copy)	0.67	0.56	0.67	0.20	0.41	0.70
APOE ε4 (≥ 1 copy)	**0.001**	0.48	**0.038**	**0.001**	**0.012**	**0.012**
SBP (10 mmHg increase)	**0.015**	0.12	**0.045**	**0.003**	**0.036**	**0.033**
DBP (10 mmHg increase)	0.11	0.21	0.23	0.34	0.29	0.45

Abbreviations: ICH = Intracerebral Hemorrhage, DBP = Diastolic Blood Pressure, SBP = Systolic Blood Pressure

Supplementary Table S6. Interaction analyses of APOE and systolic BP measurements for association with outcomes following ICH

Risk Factor	Post-ICH Outcomes					
	**ICH Recurrence**		**Ischemic Stroke**		**Small Vessel Ischemic Stroke**	
	**HR (95% CI)**	**p**	**HR (95% CI)**	**p**	**HR (95% CI)**	**p**
Interaction:<br>- APOE ε4<br>- SBP (10 mmHg increase)	**1.65 (1.15 - 3.44)**	**0.014**	0.69 (0.21 – 2.30)	0.55	**1.32 (1.01 – 1.73)**	**0.046**
**Risk Factor**	**Incident Dementia**		**Incident Depression**		**Gait Impairment**	
	**HR (95% CI)**	**p**	**HR (95% CI)**	**p**	**HR (95% CI)**	**p**
Interaction:<br>- APOE ε4<br>- SBP (10 mmHg increase)	**1.48 (1.05 – 2.09)**	**0.028**	1.80 0.95 – 3.42	0.074	**1.96 (1.22 - 3.14)**	**0.006**

Abbreviations: ICH = Intracerebral Hemorrhage, SBP = Systolic Blood Pressure

Supplementary Table S7. Interaction analyses of APOE and systolic BP measurements for association with post-ICH outcomes among patients who did not experience ICH or ischemic stroke during follow-up.

**Risk Factor**	**Post-ICH Outcomes**					
	**Incident Dementia**		**Incident Depression**		**Gait Impairment**	
	**HR (95% CI)**	**p**	**HR (95% CI)**	**p**	**HR (95% CI)**	**p**
Interaction:<br>- APOE ε4<br>- SBP (10 mmHg increase)	1.59<br>(1.08 – 2.33)	**0.018**	1.77<br>0.97 – 3.23	0.064	**1.85**<br>**(1.05 - 3.27)**	**0.035**

Abbreviations: ICH = Intracerebral Hemorrhage, SBP = Systolic Blood Pressure

Supplementary Table S8. APOE Association with Risk for Long-term Poor Outcome following ICH, Stratified by Hypertension Severity.

Patient Group Characteristics	n	HR	95% CI	p
**Normal BP**				
- *APOE* ε4: 0 copies	61	Ref.	Ref.	Ref.
- *APOE* ε4: ≥ 1 copy	14	1.10	0.87 – 1.39	0.43
**Elevated BP**				
- *APOE* ε4: 0 copies	120	Ref.	Ref.	Ref.
- *APOE* ε4: ≥ 1 copy	27	1.48	1.06 – 2.07	**0.025**
**Hypertension Stage 1**				
- *APOE* ε4: 0 copies	174	Ref.	Ref.	Ref.
- *APOE* ε4: ≥ 1 copy	35	1.79	1.18 – 2.71	**0.006**
**Hypertension Stage 2**				
- *APOE* ε4: 0 copies	239	Ref.	Ref.	Ref.
- *APOE* ε4: ≥ 1 copy	46	2.05	1.21 – 3.46	**0.008**

Supplementary Table S9. Risk for Long-term Poor Outcome following ICH, Stratified by APOE Genotype and Hypertension Severity.

Patient Group Characteristics	n	HR	95% CI	p
**Normal BP**				
- *APOE* ε4: 0 copies	61	Ref.	Ref.	Ref.
- *APOE* ε4: ≥ 1 copy	14	0.88	0.60 - 1.30	0.53
**Elevated BP**				
- *APOE* ε4: 0 copies	120	1.13	0.84 - 1.53	0.43
- *APOE* ε4: ≥ 1 copy	27	1.97	1.17 - 3.31	**0.011**
**Hypertension Stage 1**				
- *APOE* ε4: 0 copies	174	2.47	1.16 - 5.27	**0.021**
- *APOE* ε4: ≥ 1 copy	35	2.85	1.32 - 6.13	**0.008**
**Hypertension Stage 2**				
- *APOE* ε4: 0 copies	239	2.69	1.38 - 5.24	**0.004**
- *APOE* ε4: ≥ 1 copy	46	3.33	1.33 - 8.33	**0.011**

Supplementary Table S10. Improved Model Specification for Post-ICH Outcomes Resulting from Incorporation of Interaction Effects between APOE Genotype and Systolic Blood Pressure Measurements

**Model**	**Post-ICH Outcomes**					
	**ICH Recurrence**	**Ischemic Stroke**	**Small Vessel Ischemic Stroke**	**Incident Dementia**	**Incident Depression**	**Gait Impaired**
Basic model *:<br>- Demographics<br>- Medical History<br>- ICH Location<br>- Medication Exposures	0.69	0.59	0.64	0.70	0.68	0.57
Basic model<br>+ APOE	0.72	0.62	0.66	0.74	0.70	0.64
Basic model<br>+ SBP	0.75	0.68	0.71	0.75	0.73	0.66
Basic model<br>+ APOE<br>+ SBP	0.83	0.70	0.73	0.79	0.78	0.75
Basic model<br>+ APOE<br>+ SBP<br>+ APOE / SBP Interaction	**0.88**<br>(**p = 0.011**)**	0.71<br>(p = 0.39)**	0.76<br>(p = 0.18)**	**0.85**<br>(**p = 0.009**)**	**0.81**<br>(**p = 0.047**)**	**0.80**<br>**(p = 0.012)****

Table present model specification performances for post-ICH outcomes (quantified using Harrell's C), depending on variables considered for analysis. Modeling of the APOE / SBP interaction results in statistically significant improvement in model specification (compared to a model including both variables, but without an interaction term) in four out of six considered outcomes.

* Basic model includes the following variables: self-reported race / ethnicity, history of prior ICH (lobar and/or nonlobar), educational level, ICH location, antiplatelet agent use and warfarin use.

** For comparison with presented in row immediately above, including APOE and HTN, but no interaction modeling

Abbreviations: ICH = Intracerebral Hemorrhage, SBP = Systolic Blood Pressure

REFERENCES

1. Harrison JK, Fearon P, Noel-Storr AH, McShane R, Stott DJ, Quinn TJ. Informant Questionnaire on Cognitive Decline in the Elderly (IQCODE) for the diagnosis of dementia within a secondary care setting. *Cochrane Database Syst Rev* 2015; **3**: CD010772.
2. Harrison JK, Fearon P, Noel-Storr AH, McShane R, Stott DJ, Quinn TJ. Informant Questionnaire on Cognitive Decline in the Elderly (IQCODE) for the diagnosis of dementia within a general practice (primary care) setting. *Cochrane Database Syst Rev* 2014; **7**: CD010771.
3. Barber M, Stott DJ. Validity of the Telephone Interview for Cognitive Status (TICS) in post-stroke subjects. *International journal of geriatric psychiatry* 2004; **19**(1): 75-9.
4. Almeida OP, Almeida SA. Short versions of the geriatric depression scale: a study of their validity for the diagnosis of a major depressive episode according to ICD-10 and DSM-IV. *International journal of geriatric psychiatry* 1999; **14**(10): 858-65.
