# Supplementary material for: APOE genotype, hypertension severity and outcomes after intracerebral haemorrhage
Source: Brain Commun. 2019 Sep 14;1(1):fcz018. doi: 10.1093/braincomms/fcz018 (PMC7425529; doi:10.1093/braincomms/fcz018)
Supplement: fcz018_Supplementary_Materials [file fcz018_supplementary_materials.zip › fcz018_Supplementary_material_Literature_Search_Summary.pdf]

Authorship	Year	Citation	Sample Size	Study Location	Format	Study of Primary ICH?	APOE Genotype Available?	Longitudinal Blood Pressure Data Available?	SVD Outcomes Data Available?	Overlapping with Current Study?
Garcia et.al.	1999	J Neurol. 1999 Sep;246(9)830-4	245	Lisbon, Portugal	Case Control	Yes	Yes	No	No	No
Rosand et.al.	2000	Neurology. 2000 Oct 10;55(7)947-51	107	MGH - Boston, MA	Case Control	Yes	Yes	No	No	Yes
Woo et.al.	2002	Stroke. 2002 May;33(5)1190-5	554	Greater Cincinnati/ Northern Kentucky Region	Case Control	Yes	Yes	No	No	No
McCarron et.al.	2003	Acta Neurol Scand. 2003 Feb;107(2)106-9	176	Belfast, UK	Cohort Study	Yes	Yes	No	No	No
Flibotte et.al.	2003	10.1212/01.WNL.0000138428.40673.83	183	MGH - Boston, MA	Cohort Study	Yes	Yes	No	No	Yes
Greenberg et.al.	2004	Stroke. 2004 Jun;35(6):1415-20. Epub 2004 Apr 8	94	MGH - Boston, MA	Cohort Study	Yes	No	No	Yes - cognitive decline	Yes
Smith et.al.	2004	Neurology. 2004 Nov 9;63(9)1606-12	182	MGH - Boston, MA	Cohort Study	Yes	Yes	No	Yes - recurrence	Yes
Woo et.al.	2004	Stroke. 2004 Jun;35(6)1360-4. Epub 2004 Apr 15	554	Greater Cincinnati/ Northern Kentucky Region	Case Control	Yes	Yes	No	No	No
Woo et.al.	2005	Stroke. 2005 Sep;36(9)1874-9. Epub 2005 Aug 11	520	Greater Cincinnati/ Northern Kentucky Region	Case Control	Yes	Yes	No	No	No
Seifert et.al.	2006	Cerebrovasc Dis. 2006;21(4)266-70. Epub 2006 Jan 27.	193	Graz, Austria	Cohort Study	Yes	Yes	No	No	No
Saloheimo et.al.	2006	Stroke. 2006 Feb;37(2)487-91. Epub 2005 Dec 22.	346	Finland	Cohort Study	Yes	No	No	Yes - recurrence	No
Masada et.al.	2007	Brain Nerve. 2007 Feb;59(2):165-8	12	Japan	Case Control	Yes	No	No	Yes - depression	No
Marti-Fabregas et.al.	2008	10.1111/j.1468-1331.2008.02254.x	60	Barcelona, Spain	Observational	Yes	No	Yes	No	No
Attems et.al.	2008	10.1007/s00415-008-0674-4	2060	Vienna, Austria	Retrospective Cohort	Yes	No	No	Yes - dementia	No
Tzourio et.al.	2008	10.1212/01.wnl.0000308819.43401.87	5671	Multicenter internationally	Randomized Control Trial	Yes	Yes	Yes	Yes - recurrence	No
Christensen et.al.	2009	10.1161/STROKEAHA.108.538967	657	International Multicenter	Randomized Control Trial	Yes	No	No	Yes - depression	No
Domingues-Montanari et.al.	2011	10.1016/j.neurobiolaging.2010.01.019	60	Barcelona, Spain	Cohort Study	Yes	Yes	No	Yes - recurrence	No
Biffi et.al.	2011	10.1016/S1474-4422(11)70148-X	865	MGH - Boston, MA	Case Control	Yes	Yes	No	No	Yes
Zhang et.al.	2012	10.1089/gtmb.2011.0103	360	China	Case Control	Yes	Yes	No	No	No
Biffi et.al.	2012	10.1212/WNL.0b013e3182452b40	680	MGH - Boston, MA	Case Control	Yes	Yes	No	No	Yes
Biffi et.al.	2012	10.1016/S1474-4422(11)70148-X	865	Multicenter across the USA	Case Control	Yes	Yes	No	No	Yes
Brouwers et.al.	2012	10.1161/STROKEAHA.112.659094	1082	MGH - Boston, MA	Retrospective Cohort	Yes	Yes	No	No	Yes
Martini et.al	2012	10.1212/WNL.0b013e318276896f	2145	Greater Cincinnati/ Northern Kentucky Region	Case Control	Yes	Yes	No	No	No
Garcia et.al.	2013	10.1016/j.jstrokecerebrovasdis.2011.06.013	183	Amiens, France	Cohort Study	Yes	No	No	Yes - dementia	No
Misra et.al.	2013	10.1016/j.jstrokecerebrovasdis.2012.02.006	242	India	Case Control	Yes	Yes	No	No	No
Woo et.al.	2013	10.1161/STROKEAHA.113.001304	2002	Greater Cincinnati/ Northern Kentucky Region	Case Control	Yes	Yes	No	No	No
Kim et.al.	2013	10.1016/j.clineuro.2012.07.010	2384	South Korea	Cohort Study	Yes	No	No	Yes - recurrence	No
Howard et.al.	2013	10.1161/STROKEAHA.111.000529	30,239	Multicenter across the USA	Cohort Study	Yes	No	No	Yes - recurrence	No
Samarasekera et.al.	2015	10.1161/STROKEAHA.114.007953	128	United Kingdom	Cohort Study	Yes	No	No	Yes - recurrence	No
Benedictus et.al.	2015	10.1161/STROKEAHA.115.010200	167	Amsterdam, The Netherlands	Cohort Study	Yes	No	No	Yes - cognitive decline	No
Koivunen et.al.	2015	10.1111/ane.12367	336	Helsinki, Finland	Cohort Study	Yes	No	No	Yes - depression, cognitive decline	No
Raffeld et.al.	2015	10.1212/WNL.0000000000001790	363	MGH - Boston, MA	Cohort Study	Yes	Yes	No	Yes - recurrence	Yes
Biffi et.al.	2015	10.1001/jama.2015.10082	1145	MGH - Boston, MA	Observational	Yes	No	Yes	Yes - recurrence	Yes
Kuramatsu et.al.	2015	10.1001/jama.2015.0846	1176	Germany	Retrospective Cohort	Yes	No	No	Yes - recurrence	No
Radholm et.al.	2015	10.1093/ageing/afu198	2839	Multicenter internationally	Randomized Control Trial	Yes	No	No	Yes - depression	No
Phuah et.al.	2016	10.1212/NXG.0000000000000081	129	MGH - Boston, MA	Retrospective Cohort	Yes	Yes	No	No	Yes
Montanola et.al.	2016	10.1007/s12017-015-8381-7	262	Barcelona, Spain	Case Control	Yes	Yes	No	No	No
Roongpiboonsopit et.al.	2016	Neurology. 2016 Nov 1;87(18)1863-1870. Epub 2016 Sep 30.	474	MGH - Boston, MA	Cohort Study	Yes	Yes	No	Yes - recurrence	Yes
Krishnan et.al.	2016	10.1016/j.jstrokecerebrovasdis.2016.01.010	629	International Multicenter	Randomized Control Trial	Yes	No	No	Yes - mood, cognition	No
Biffi et.al.	2016	10.1001/jamaneurol.2016.0955	738	MGH - Boston, MA	Cohort Study	Yes	Yes	No	Yes - dementia only	Yes
Woo et.al.	2016	10.1212/WNL.0000000000002449	1681	Multicenter across the USA	Case Control	Yes	No	No	Yes - gait	No
Raposo et.al	2017	10.1212/WNL.0000000000004228	33	Toulouse, France	Case Control	Yes	Yes	No	No	No
Stern-Nezer et.al.	2017	10.3233/NRE-171470	89	Stanford, CA	Cohort Study	Yes	No	No	Yes - depression	No
Teo et.al.	2017	10.1016/j.wneu.2017.06.015	109	Hong Kong, China	Retrospective Cohort	Yes	No	Yes	Yes - recurrence	No
Planton et.al.	2017	https//.org/10.1371/journal.pone.0178886	142	Toulouse, France	Case Control	Yes	No	No	Yes - depression, cognitive decline	No
Boulouis et.al.	2017	10.1016/j.jns.2017.07.015	229	MGH - Boston, MA	Cohort Study	Yes	No	No	Yes - recurrence	Yes
Charidimou et.al.	2017	10.1212/WNL.0000000000004665	313	MGH - Boston, MA	Cohort Study	Yes	Yes	No	Yes - recurrence	Yes
Renard et.al.	2018	10.3233/JAD-180269	48	France	Cohort Study	Yes	Yes	No	No	No
Rodriguez-Torres et.al.	2018	10.1212/WNL.0000000000005729	2291	MGH - Boston, MA	Case Control	Yes	No	Yes	Yes - recurrence	Yes
Sawyer et.al.	2018	10.1212/WNL.0000000000005908	3567	Multicenter across the USA	Case Control	Yes	Yes	No	No	No
